# Observations on a Neuralgia of the Facial Nerve

**Published:** 1818-04

**Authors:** M. Lemercier

**Affiliations:** Mayenne.


*For the London Medical and Physical Journal.*

### *Observations on a Neuralgia of the Facial Nerve;* by M. Lemercier, M.D. at Mayenne.

[From our Correspondent at Paris.]

THE Marquis of St. Souplet, sixty-one years old, of a sanguine temperament, good constitution, a mild disposition, with kind and pleasing manners, in good health, and subject to no particular complaint, was seized (the 26th of November, 1817) with a very sharp lancing pain in the right ear and cheek. This violent pain lasted about five minutes, and then ceased as suddenly as it had come on. He attributed it to the cold and damp, did nothing but stuff his ear with cotton, and keep himself warm the rest of the day: in the evening, and during the night, the pain came on by paroxysms, and was more and more intense.

On the 27th, he remained free till the evening, when the pains were even more frequent and violent than the day before. Nevertheless, during the interval, he went into company; and, in my presence, was suddenly seized with cutting pains in the ear and cheek, which, after ten minutes of the greatest suffering, went off abruptly. The Marquis continued his game, and told me he felt nothing whatever.

On examining the cheek, I could perceive nothing in particular. The same night he suffered considerably at different times, and hardly slept at all; yet, felt no inconvenience between the fits, but always dreading their return.

On the 28th, in the morning, he sent for me, having suffered severely during the night. I examined the inside of the ear attentively, without perceiving any irritation. I touched the cheek and surrounding parts, without giving the least pain, and without perceiving any swelling or redness any where. I advised frequent injections into the ear with a decoction of poppy-head; but the pains returned at intervals during the day and night. The next day, I added to each injection twelve drops of liquid laudanum: the sufferings were the same as the preceding days, and the paroxysms were even more frequent, and lasted longer, coming on suddenly, as before. The pains seemed to arise from before the ear, and, diverging internally, spread all at once over the muscles of its posterior part, to the concha, the parotid gland, between that and the condyle of the lower jaw; over the temple, the lower eyelid, the cheek, the teeth; above and below the chin, the lips, and the *ala nasi* of that side. All the muscular fibres of those parts were simultaneously agitated with violent spasms. To deaden this cruel pain, the patient used to press his head with his hands, or shake it strongly. When these shooting tearing pains had lasted about half an hour, they ceased entirely; and there was neither pain, nor redness, nor swelling, nor any particular feel even on pressing the parts hardly with the finger. I prescribed several vapour-baths every day; a repetition of the injection, with a double dose of laudanum; and the cheek to be rubbed with sulphuric ether. That night the spasms were less violent, but the lancing pains continued.

The 30th, in the morning, I ordered two leeches behind the ear, which drew a great deal of blood. The vapour-baths and frictions, with ether, were continued. In the evening, the fits came on three or four times, but were less sharp; and, from that time, the patient gradually recovered, with only the occasional use of ether on slight returns.

May not this case serve to show, that tic douloureux is sometimes the effect of inflammation in the nerves of those parts?

*Paris; March* 1818.

